# Characteristics of the prehospital phase of adult emergency department patients with an infection: A prospective pilot study

**DOI:** 10.1371/journal.pone.0212181

**Published:** 2019-02-07

**Authors:** Gideon H. P. Latten, Lieke Claassen, Marnix Jonk, Jochen W. L. Cals, Jean W. M. Muris, Patricia M. Stassen

**Affiliations:** 1 Emergency Department, Zuyderland Medisch Centrum, Heerlen, The Netherlands; 2 Department of Family Medicine, Maastricht University, School of CAPHRI, Maastricht, The Netherlands; 3 Department of Internal Medicine, Division General Medicine, Section Acute Medicine, Maastricht University, School of CAPHRI, Maastricht, The Netherlands; Sunnybrook Research Institute, CANADA

## Abstract

**Objective:**

Research on serious infections/sepsis has focused on the hospital environment, while potentially the most delay, and therefore possibly the best opportunity to improve quality of care, lies in the prehospital setting. In this study we investigated the prehospital phase of adult emergency department (ED) patients with an infection.

**Methods:**

In this prospective pilot study all adult (≥18y) patients with a suspected/proven infection, based on the notes in the patient’s ED chart, were included during a 4-week period in 2017. Prehospital course, ED findings, presence of sepsis and 30-day outcomes were registered.

**Results:**

A total of 440 patients were identified, with a median symptom duration before ED visit of 3 days (IQR 1–7 days). Before arrival in the ED, 23.9% of patients had used antibiotics. Most patients (83.0%) had been referred by a general practitioner (GP), while 41.1% of patients had visited their GP previously during the current disease episode. Patients referred by a GP were triaged as high-urgency less often, while vital parameters were similar. Emergency Medical Services (EMS) transported 268 (60.9%) of patients. Twenty-two patients (5.0%) experienced an adverse outcome (30-day all-cause mortality and/or admission to intensive care).

**Conclusions:**

Patients with a suspected infection had symptoms for 3 (IQR 1–7) days at the moment of presentation to the ED. During this prehospital phase patients often had consulted, and were treated by, their GP. Many were transported to the ED by EMS. Future research on severe infections should focus on the prehospital phase, targeting patients and primary care professionals.

## Introduction

One of the challenges for physicians is to timely recognize patients with an infection who are at risk of developing sepsis. Similar to myocardial infarction and stroke, mortality in sepsis patients increases with delayed treatment.[1,2] Early recognition and treatment of patients at risk therefore provide an opportunity to improve outcome.

Over the past years, timely recognition and treatment of patients with sepsis has improved. However, research has focused on sepsis within the hospital and not on the prehospital professionals: emergency medical services (EMS) and general practitioners (GPs).[3,4] To our knowledge, the prehospital phase of ED patients with a suspected infection has not yet been investigated before. This phase however, could potentially include most delay and may therefore possibly be the best phase to focus on when improving quality of care for sepsis patients.

In this prospective pilot study, we investigate the prehospital phase of adult ED patients with a suspected infection. We specifically aim to investigate the duration of symptoms, number of GP contacts in the current disease episode, use of antibiotics, adverse outcomes (30-day all-cause mortality and/or intensive care unit (ICU) admission) and referral pathway (involvement of GP and/or EMS).

## Methods

### Design and setting

This prospective pilot study took place during a 4-week period between 23 January and 19 February 2017 in Zuyderland Medical Centre, a large teaching hospital located in Heerlen, the Netherlands. Yearly, approximately 35,000 patients are assessed and treated in our ED by either emergency physicians or residents of other specialties. The majority of patients are referred by a GP. In the Netherlands, these are well trained primary care physicians, who provide the first step in emergency care 24/7 from their practices or out-of-hours services. The remaining patients contact the EMS or visit the ED on their own initiative. All patients are triaged by a dedicated triage nurse, using the Manchester Triage System.[5] After diagnosis and treatment in the ED, patiens are either discharged home, or admitted to the hospital (regular wards, specific medium care units (e.g. brain care unit, cardiac care unit), or ICU).

We used the Strengthening the Reporting of Observational Studies in Epidemiology guidelines for reporting this observational study.[6] The study was reviewed and approved by the medical ethics committee of Zuyderland (METC-Z nr. 16-N-202).

### Patients

All ED patients aged 18 years or older were included if they had a suspected or proven infection, based on signs and symptoms mentioned in the referral letter and/or the patients’ ED chart. All charts were checked manually to find evidence of suspicion of infection. One investigator (MJ) screened all patients for inclusion in the study. If it was unclear whether a patient should be included, a second investigator (GL or LC) was consulted and consensus was reached. To avoid any errors, a random sample of 10% of all data were double-checked by a second investigator. Patients visiting the ED more than once during the study period were included at their initial visit only.

### Data collection

Patient data were collected using a Case Report Form (CRF) comprising data from the patient chart, including the referral letter and EMS notes. Additional information from GP or patient was requested by telephone, if necessary (i.e. use of antibiotics, previous GP consultation, and outcome). Table 1 shows the variables that were retrieved and the definitions that were used.

**Table 1:** Documented variables and used definitions.

Documented variables	
**General**	
Age	
Sex	
Comorbidities	Quantified using the Charlson Comorbidity Index (CCI)[7]
Number of medications	
**Prehospital phase**	
Duration of symptoms (days)	
Use of antibiotics	At moment of presentation to the ED and in the preceding 30 days
Previous GP consultation during current disease episode	Starting from the first day of symptoms and not including GP consultation on the day of ED referral
**Ambulance phase**	
Mode of transportation to hospital	Ambulance or other means of transportation
EMS urgency	Assigned by the ambulance dispatch center following the Netherlands Triage Standard (NTS).[8] A1: most urgent category, life-threatening situation; A2: urgent but not life-threatening; B: non-urgent conditions.
**Emergency department phase**	
Referral to the ED	Current visit: referred by GP or not?
Level of triage	Determined using the Manchester Triage System (MTS).[5] Assessment necessary: Red: immediately, orange: ≤10 minutes, yellow: ≤60 minutes, green: ≤120 minutes and blue: ≤240 minutes. We combined the red and orange urgency as ‘high urgency’ and the yellow, green and blue urgency as ‘low urgency’.
ED vital parameters	Lowest systolic blood pressure (mmHg) Lowest diastolic blood pressure (mmHg) Mean arterial pressure (MAP, mmHg) Highest heart rate (beats per minute, bpm) Lowest oxygen saturation (%) Highest respiratory rate (/minute) Most abnormal temperature (°C) Lowest Glasgow Coma Scale (GCS)
**Patient outcomes**	
Focus of infection	Respiratory, urogenital, abdominal, skin, cardiovascular, central nervous system, unknown
Admission to hospital	all departments
ICU admission	
Length of stay in the hospital (LOS)	
30-day all-cause mortality	
**Definitions**	
Sepsis	Suspected or proven infection and the presence of two or more Systemic Inflammatory Response Syndrome (SIRS) criteria and/or quick Sepsis-related Organ Failure Assessment (qSOFA) criteria [9,10] We choose to use both the old (systemic inflammation (≥2 SIRS criteria) and infection) and the new definition of sepsis (qSOFA ≥2 or SOFA score>2), because the new definition has just been introduced and research comparing both is still ongoing.[11–13] Primary care and ED professionals can use SIRS and qSOFA as screening tools for sepsis in contrast to the SOFA score.
SIRS	Temperature >38°C or <36°C, heart reate >90/min, respiratory rate >20/min or PaCO_2_ <4.3kPa (32 mmHg), white blood cell count >12000/mm^3^ or <4000/mm^3^ or >10% immature bands
qSOFA	Respiratory rate ≥22/min, altered mentation, systolic blood pressure ≤100 mmHg[9]
SOFA	0–24 points, depending on PaO_2_/FiO_2_ ratio, platelet count, bilirubin, MAP, administration of vasopressors (type and dose), Glasgow Coma Scale and serum creatinine or urine output[9]
Sepsis severity	Sepsis: sepsis in the absence of severe sepsis or septic shock Severe sepsis: sepsis complicated by organ failure[10] Septic shock: sepsis with a mean arterial pressure (MAP) <60 mmHg, despite adequate fluid resuscitation
Adverse outcomes	All-cause mortality within 30 days and/or admission to ICU
Focus of infection at discharge	Focus at the moment of discharge from the hospital or from the ED (not-admitted patients)

### Analysis

Descriptive analysis was performed, using the variables in Table 1, to provide insight in the prehospital and ED phase of patients with an infection. GP-referred patients were compared with unreferred patients, regarding prehospital and ED characteristics, presence of sepsis and outcome. In addition, patients with an adverse outcome (30-day all-cause mortality and/or ICU admission) were compared with those without adverse outcome.

### Statistical methods

All statistical analyses were performed using IBM SPSS statistical software version 21 (Chicago, Illinois, USA). Continuous data were reported as means with standard deviation (SD) and compared using Students’ T test, or as medians with interquartile ranges (IQR) and compared using the Mann Whitney U test. We reported categorical data as absolute numbers and as valid percentages (to correct for missing data); they were compared using chi-square or Fisher exact tests. Differences in mortality were calculated using the Kaplan-Meier method and the log-rank test. A P value <0.05 was considered statistically significant.

## Results

### Participants

During the inclusion period, 2163 adult patients visited our ED; 440 patients (20.3%) had a suspected or proven infection.

### Characteristics of patients with a proven/suspected infection

The mean age was 67 years and median CCI score was 2 (IQR 1–3, Table 2). The median duration of symptoms before ED arrival was 3 days (IQR 1–7 days) and 83.0% of patients were referred by a GP. In the period preceding the ED visit, 41.1% had already consulted their GP at least once, while 32.7% had used antibiotics in the preceding 30 days. Patients were transported by ambulance in 60.9%, most commonly as EMS urgency A2 (45.9%). Patients were triaged as high urgency in the ED in 25.9%: ≥2 SIRS-criteria were present in 58.9%, and ≥2 qSOFA-criteria in 12.3% of patients. Eighty percent of patients were admitted to the hospital.

**Table 2:** Baseline characteristics and outcomes of patients with a suspected/proven infection in the ED and a comparison between GP-referred and unreferred patients.

	Total (n = 440)	N	GP-referred patients (n = 365 (83.0%))^a^	Unreferred patients (n = 71 (16.1%))^a^	P value
**General**					
Age (years)	67 (±18)	440	67 (±18)	66 (± 15)	0.63
Male	218 (49.5%)	440	178 (48.8%)	37 (52.1%)	0.61
Charlson Comorbidity Index	2 (1–3)	440	2 (1–3)	2 (0–4)	0.29
Number of medications	6 (±4)	440	5 (±4)	6 (±4)	0.71
**Prehospital phase**					
Duration of symptoms in days	3 (1–7)	440	3 (1–7)	3 (2–4)	0.41
Visited GP previously	181 (41.1%)	440	159 (43.6%)	21 (29.6%)	0.03*
Use of antibiotics in past 30 days	144 (32.7%)	440	126 (34.5%)	17 (23.9%)	0.08
Current use of antibiotics	105 (23.9%)	440	92 (25.2%)	12 (16.9%)	0.13
**Ambulance phase**					
Transport by ambulance	268 (60.9%)	440	216 (59.2%)	48 (67.6%)	0.18
EMS urgency		268			<0.001*
A1	65 (24.3%)		40 (18.5%)	25 (52.1%)	
A2	123 (45.9%)		104 (48.1%)	18 (37.5%)	
B	73 (27.2%)		68 (31.5%)	2 (4.2%)	
**Emergency Department phase**					
High urgency triage (red/orange)	114 (25.9%)	440	87 (23.8%)	25 (35.2%)	0.04*
Systolic blood pressure–mmHg	135 (±28)	410	135 (±28)	135 (±27)	0.93
Diastolic blood pressure–mmHg	74 (±17)	410	74 (±18)	74 (±17)	0.87
Mean arterial pressure–mmHg	94 (±18)	410	94 (±18)	94 (±18)	0.97
Heart rate–bpm	97 (±23)	429	97 (±23)	100 (±24)	0.25
Oxygen saturation—%	95 (92–97)	426	95 (92–97)	95 (91–97)	0.31
Respiratory rate/min	20 (16–24)	434	20 (16–24)	20 (16–24)	0.51
Temperature–°C	37.5 (37.0–38.3)	432	37.5 (37.0–38.2)	37.7 (36.9–38.7)	0.40
Glasgow Coma Scale	15 (15–15)	440	15 (15–15)	15 (15–15)	0.82
SIRS ≥2	259 (58.9%)		209 (57.3%)	47 (66.2%)	0.16
qSOFA ≥2	54 (12.3%)		43 (11.8%)	10 (14.1%)	0.59
SOFA ≥2	240 (54.5%)		202 (55.3%)	38 (53.5%)	0.78
SOFA score	2 (1–3)		2 (1–3)	2 (0–3)	0.82
**Sepsis severity**					0.41
Sepsis (no severe sepsis or shock)	102 (23.2%)		79 (21.6%)	21 (29.6%)	
Severe sepsis	158 (35.9%)		131 (35.9%)	26 (36.6%)	
Septic shock	5 (1.1%)		4 (1.1%)	1 (1.4%)	
No sepsis	175 (39.8%)		151 (41.4%)	23 (32.4%)	
**Outcome**					
Admission to hospital	352 (80.0%)	440	284 (77.8%)	64 (90.1%)	0.02*
ICU admission	15 (3.4%)	440	7 (1.9%)	8 (11.3%)	<0.001*
Length of stay (LOS)–days	6 (4–11)	440	6 (4–11)	6 (3–11)	0.65
30-day mortality	10 (2.3%)	440	7 (1.9%)	3 (4.2%)	0.95
Focus of infection at discharge		440			0.44
Respiratory	269 (61.1%)		229 (62.7%)	38 (53.5%)	
Urogenital	55 (12.5%)		39 (10.7%)	14 (19.7%)	
Abdominal	46 (10.5%)		39 (10.7%)	7 (9.9%)	
No infection^b^	11 (2.5%)		10 (2.7%)	1 (1.4%)	
Skin	9 (2.0%)		7 (1.9%)	2 (2.8%)	
Cardiovascular	4 (0.9%)		4 (1.1%)	0	
Central nervous system	1 (0.2%)		1 (0.3%)	0	
Other or focus unknown	45 (10.2%)		36 (9.9%)	9 (12.7%)	

Values: n (%), mean (±SD), or median (IQR)

^a^ for 4 patients referral pathway could not be retrieved (GP-referred or unreferred)

Abbreviations: GP, general practitioner; CCI, Charlson Comorbidity Index; EMS, emergency medical services

^b^ patients who had a suspected infection in the ED, but were diagnosed with other pathology after admission (eg. pancreatitis, intoxication)

### Comparison of referred with unreferred patients

In total, 365 (83.0%) patients had been referred by a GP (Table 2). General characteristics did not differ between the two groups. Median duration of symptoms was 3 days in both groups, but referred patients more often had visited their GP earlier during the current disease episode (43.6 vs. 29.6%, p = 0.03) and more often had used antibiotics, although this difference was not significant (34.5 vs. 23.9%, resp., p = 0.08). Referred patients were less often triaged as high urgency by the EMS (A1 18.5 vs. 52.1%, p<0.001) and by the ED (23.8 vs. 35.2%, p<0.05) than unreferred patients. Vital parameters and the proportion of patients with SIRS/(q)SOFA scores ≥2 did not differ between the two groups. Referred patients were admitted to the hospital and ICU less often (77.8 vs. 90.1%, p<0.05 and 1.9 vs. 11.3%, p<0.001). All-cause 30-day mortality was higher in the referred group, although this difference was not significant (1.9 vs. 4.2%, p = 0.24).

### Comparison of patients with and without adverse outcomes

Twenty-two (5.0%) patients experienced in total 25 adverse outcomes: 15 (3.4%) were admitted to the ICU and 10 (2.3%) patients died (Table 3). There were no significant differences in general characteristics, but patients with an adverse outcome were less often referred by a GP (59.1 vs. 84.2%, p = 0.001) and were considered more urgent by both EMS (A1: 52.6 vs. 22.1%, resp., p<0.05) and ED (highly urgent in 72.7 vs. 23.4%, resp., p<0.001). The number of patients with ≥2 SIRS criteria and vital parameters did not differ, except for the respiratory rate, which was higher in the adverse outcome group (22.5 vs. 20.0, p = 0.02). In patients with adverse outcomes, both qSOFA and SOFA scores were more often ≥2 than in the adverse outcome group (qSOFA 36.4 vs. 11.0%, p<0.001; SOFA 81.1 vs. 53.3%, p = 0.01).

**Table 3:** Comparison between patients with and without an adverse outcome.

	Adverse outcome^a^ (n = 22)	No adverse outcome^a^ (n = 418)	N	P value
**General**				
Age (years)	70.1 (±13.7)	67.1 (±17.9)	440	0.44
Male	10 (45.5%)	208 (49.8%)	440	0.69
Charlson Comorbidity Index	2 (1–3.25)	2 (1–3)	440	0.38
Number of medications	7 (±3)	5 (±4)	440	0.17
**Prehospital phase**				
Duration of symptoms in days	2.5 (1–4)	3 (1–7)	440	0.23
Visited GP previously	9 (40.9%)	172 (41.1%)	440	0.98
Use of antibiotics in past 30 days	6 (27.3%)	138 (33.0%)	440	0.58
Current use of antibiotics	5 (22.7%)	100 (23.9%)	440	0.90
**Ambulance phase**				
Transport by ambulance	19 (86.4%)	249 (59.6%)	440	0.01*
EMS urgency			440	0.01*
A1	10 (52.6%)	55 (22.1%)		
A2	7 (36.8%)	116 (46.6%)		
B	2 (10.5%)	71 (28.5%)		
**Emergency Department phase**				
Referred by GP–current ED visit	13 (59.1%)	352 (84.2%)	440	0.001
High urgency triage (red/orange)	16 (72.7%)	98 (23.4%)	440	<0.001*
Systolic blood pressure–mmHg	127.6 (±43.7)	134.9 (±26.6)	410	0.47
Diastolic blood pressure–mmHg	67.9 (±22.3)	74.0 (±16.7)	410	0.23
Mean arterial pressure–mmHg	87.8 (±27.5)	94.3 (±17.7)	410	0.30
Heart rate–bpm	102.6 (±36.6)	97.2 (±21.8)	429	0.52
Oxygen saturation—%	92.0 (90.0–97.0)	95.0 (92.0–97.0)	426	0.09
Respiratory rate/min	22.5 (19.0–30.5)	20.0 (16.0–24.0)	434	0.02*
Temperature–°C	37.4 (36.2–38.0)	37.6 (37.0–38.4)	432	0.23
Glasgow Coma Scale	15.0 (12.8–15.0)	15.0 (15.0–15.0)	440	<0.001*
SIRS ≥2	11 (50.0%)	248 (59.3%)		0.39
qSOFA ≥2	8 (36.4%)	46 (11.0%)		<0.001*
SOFA ≥2	18 (81.8%)	223 (53.3%)		0.01*
SOFA (score)	4 (3–5)	2 (0–3)		<0.001*
**Sepsis severity**				<0.001
Sepsis (no severe sepsis or shock)	0	102 (24.4%)		
Severe sepsis	9 (40.9%)	149 (35.6%)		
Septic shock	3 (13.6%)	2 (0.5%)		
No sepsis	10 (45.5%)	165 (39.5%)		
**Outcome**				
Admission to hospital	22 (100%)	330 (78.9%)	440	0.02*
Length of stay (LOS)–days	10 (5.8–13.3)	6 (4–11)	440	0.03*

^a^ Adverse outcome defined as 30-day all-cause mortality and/or ICU admission

## Discussion

To the best of our knowledge, no other study has prospectively investigated the prehospital phase of ED patients with a suspected infection. In a median period of 3 days before visiting the ED, many (41.1%) patients had prior contact with their GP, and 23.9% had already used antibiotics. For the actual ED visit, GPs referred most patients (83.0%) and many were transported by ambulance (60.9%). Between referred and unreferred patients, no differences in general characteristics, vital parameters or sepsis criteria were found. However, referred patients were less often placed in a high triage category or admitted to either the hospital (77.8 vs. 90.1%, p = 0.02) or ICU (1.9 vs 11.3%, p<0.001). Patients who experienced an adverse outcome (5.0%) had the same duration of symptoms, number of GP contacts and prior use of antibiotics as those without an adverse outcome.

Our study shows that for most ED patients with an infection, the acute care chain starts with a contact with the GP and transport by EMS. These findings suggest that the acute care chain offers a window of opportunity that allows for a good start of treatment. It is probable that the prehospital phase is important and that it influences choices that are made in the ED, although no studies have taken this phase into account when evaluating sepsis.

Selecting those in need of hospital care is one of the challenges GPs have to deal with. In our study, the majority (83.0%) of patients was referred by a GP. These patients were considered less urgent by the EMS and tha ED than unreferred patients. An explanation could be that unreferred patients accurately assessed their situation as highly urgent and called for help (EMS). One study investigated why GPs refer patients with an infection. General patient appearance, gut feeling and patient history turned out to be most important for the decision whether or not to refer.[14] Our finding that the respiratory rate was higher in patients with an adverse outcome, may suggest that including this vital parameter in this decision-making process could be useful. In 2016, a NICE guideline provided recommendations for GPs when to refer patients with suspected sepsis to the hospital.[15] The guideline committee has recommended that an evaluation of implementation of the guideline should be performed.[16] As far as we know, this has not been done yet. It would be interesting to investigate whether the selection process and the treatment started by GPs is optimal. For this analysis, data on symptoms, vital signs and treatment in the GP-phase must be retrieved. Further, a way of assessing the accuracy of the referral policy and prehospital treatment must be developed: just right/too early/too late. Consensus meetings could contribute to this assessment, but interviewing patients should also be considered. Their behaviour probably influences treatment of the infection (eg. patient delay) and their assessment of care is important.

It should be noted that EMS are a key player in the acute care chain as well. EMS staff decides what route their patients follow. This is important since documentation of sepsis by EMS could be further improved, whereas patients who are recognized receive appropriate care sooner when they subsequently arrive in the ED.[3,4,17–19]

Future research may focus on patient education, appropriate triage, early treatment, including ED referral, and the use of point-of-care testing (POCT), such as lactate and/or CRP.

### Limitations

Patients were included when an infection was suspected/proven. It is possible that some patients were missed when an infection was not appropriately documented or recognized in the ED. Furthermore, sometimes vital parameters were missing. Missing data were retrieved by asking patients and retrieving referral handover information. Information may have been incomplete, but this loss of information was random and therefore has not influenced our results. In addition, the organization of acute health care in the Netherlands probably differs from that in other, which makes the number of self-referrals relatively small. This organisation of care can make extrapolation to other countries difficult. Finally, our cohort was included during a flu episode, which may have influenced our patient characteristics: 20% of patients with an infection seems high. We therefore evaluated ED visits in other months of 2017 and found an equal number of ED visits because of infections. An explanation for this high proportion of infections is that our GPs prevent ED visits for minor complaints, like small trauma.

In conclusion, patients with an infection in our ED had a median symptom duration of 3 days, regardless of the way of referral. One in four patients already used antibiotics and almost half of patients visited their GP once or more before they were referred. Future research should further investigate the prehospital pathway and outcomes of sepsis patients.

## Supporting information

S1 Dataset Variables included in analysis. (XLS)
